# Supplementary material for: Demographic history and population structure of Phlebotomus argentipes (Diptera: Psychodidae) complex, the leishmaniasis vector in Sri Lanka
Source: PLoS One. 2025 Dec 2;20(12):e0337428. doi: 10.1371/journal.pone.0337428 (PMC12671780; doi:10.1371/journal.pone.0337428)
Supplement: S1 Table — (DOCX) [file pone.0337428.s001.docx]

**Supplementary Materials:**

Table S1- *Phlebotomus argentipes* s.l*.* sandfly collection record from five different collection sites in Sri Lanka.

| **Location ID** | **Code** | **Trapping Method** | **Total** | **Female** | **Male** | **Total collection per location** | **Number of *P. argentipes s.l.* specimens subjected to DNA extraction** | **Number of sequences** | | |
| --- | --- | --- | --- | --- | --- | --- | --- | --- | --- | --- |
|  |  |  |  |  |  |  |  | ***COI*** | ***ND4*** |  |
| Hambantota | HAM | CBNT | 56 | 9 | 47 | 75 |  | 40 | 31 |  |
|  |  | LT | 12 | 4 | 8 |  | 41 |  |  |  |
|  |  | ST | 0 | 0 | 0 |  |  |  |  |  |
|  |  | MC | 7 | 4 | 3 |  |  |  |  |  |
| Anuradhapura | ANU | CBNT | 23 | 4 | 19 | 44 |  | 25 | 27 |  |
|  |  | LT | 15 | 3 | 12 |  |  |  |  |  |
|  |  | ST | 4 | 2 | 2 |  | 27 |  |  |  |
|  |  | MC | 2 | 2 | 0 |  |  |  |  |  |
| Balangoda | BAL | CBNT | 30 | 7 | 23 | 48 |  | 31 | 29 |  |
|  |  | LT | 8 | 6 | 2 |  | 32 |  |  |  |
|  |  | ST | 5 | 2 | 3 |  |  |  |  |  |
|  |  | MC | 5 | 2 | 3 |  |  |  |  |  |
| Mirigama | MIR | CBNT | 26 | 11 | 15 | 48 |  | 28 | 21 |  |
|  |  | LT | 13 | 7 | 6 |  | 31 |  |  |  |
|  |  | ST | 3 | 1 | 2 |  |  |  |  |  |
|  |  | MC | 6 | 2 | 4 |  |  |  |  |  |
| Medirigiriya | MED | CBNT | 24 | 7 | 17 | 49 |  | 24 | 25 |  |
|  |  | LT | 14 | 5 | 9 |  | 28 |  |  |  |
|  |  | ST | 6 | 3 | 3 |  |  |  |  |  |
|  |  | MC | 5 | 4 | 1 |  |  |  |  |  |
| Total | | | | 85 | 179 | 264 | 159 | 148 | 133 |  |
