# Supplementary material for: Demographic history and population structure of Phlebotomus argentipes (Diptera: Psychodidae) complex, the leishmaniasis vector in Sri Lanka
Source: PLoS One. 2025 Dec 2;20(12):e0337428. doi: 10.1371/journal.pone.0337428 (PMC12671780; doi:10.1371/journal.pone.0337428)
Supplement: Attachment — Submitted filename: Comments_PlosOne.docx [file pone.0337428.s004.docx]

The manuscript titled "Population expansion pattern of *Phlebotomus argentipes* (Diptera: Psychodidae) complex, the leishmaniasis vector in Sri Lanka" presents population genetic analyses based on COI and ND4 sequence data generated in their previous study. The data and observations are indeed interesting and have considerable importance for understanding vector dynamics in Sri Lanka. However, in its current form, the manuscript does not effectively convey the true value and significance of the findings. Several major issues need to be addressed to improve the clarity, scientific rigor, and impact of the manuscript:

1.	Many sections of the manuscript are unnecessarily lengthy and deviate from the core data and analyses

2.	Several statements are exaggerated and should be revised to reflect a more balanced interpretation of the results.

3.	The manuscript contains redundant and sometimes contradictory statements that compromise its logical flow and coherence.

4.	Some of the cited literature is not directly relevant to the study and should be revised

5.	At several places in the manuscript, the flow of ideas is unclear, making it difficult to understand the context or relate certain statements to the preceding content.

The manuscript presents potentially valuable findings, but it requires substantial restructuring, elimination of speculative statements, tightening of narrative flow, and a more critical and scientifically sound discussion of the results.

**I have listed here my observations for consideration:**

The title could be revised for clarity and broader relevance.

Suggest using:

“Demographic history of *Phlebotomus argentipes* (Diptera: Psychodidae), the leishmaniasis vector in Sri Lanka” or “**Population dynamics**…”

**Abstract:**

- The abstract should clarify that data were reused from a previous study.
- Statements such as “across Sri Lanka” are misleading given that only five sites were sampled and out of which two showed high Fst values.
- “**consistent** genetic homogeneity” consistent can be removed
- Lines 43-44: “genetically homogenous population with substantial gene flow across Sri Lanka”
- Both homogeneity and gene flow have not been estimated in the study. It is just an indirect observation basedon fst values
- Line 46: add linearized before (Fst/1-FSt)
- Line 49: “which may reflect vector-parasite interaction” is not clear
- Conclusing staments need to repharsed and should only be based on the egentic evidence generated form this study

Write clearly a line or two about the analysis carried out (like pairwise fst, Mantle test and mismatch distribution and bayseian skyline plot analysis) and then briefly include the observations from each analyses followed by concluding statement based on the observations.

**Introduction:**

- Lines 60–62, 71–72: Multiple citations are used to support claims that are either weakly linked or unverified in those references
- Some general statements are either not novel or not necessary (Line 83).
- Phrases such as “historical analysis of recent descendants’ molecular data” are ambiguous and need clearer scientific language.
- Line 83: This is general statement and the reference cited has not analyzed this.
- Structure and flow need significant improvement to clarify rationale and research gap.

**Materials and Methods:**

- The data were not generated in this study. The first two paragraphs should be condensed into a brief statement referencing the previous work.
- Total number of samples and sequences per population should be explicitly mentioned.
- Figure 1 is redundant as already information given in previous study
- Line 113: “hence” is changing the meaning of the sentence
- Suggest performing additional analyses such as STRUCTURE, concatenated gene analysis of COI and ND4, increasing permutations for Fst estimation (Line 147) and the analysis should also be carried out for the pooled sequences from all populations for comparison.

**Results:**

- Initial nucleotide composition (Table 1) and base content discussion seem unnecessary. Suggest replacing this with some relevant information like population-wise sequence numbers, geographic distance between them, diversity indices (citing previous study).
- Lines 184-185: pairwise differences has not been estimated in this study and please replace "examined localities" with "study populations").
- Check highest Fst values, Table 2 suggests MIR–BAL for ND4, not MED–HAM.
- Provide correlation coefficients and p-values with Mantel test (Line 191).
- Table 2 and figure 4 legends require better wording (e.g., Table 2 legend: “Genetic differentiation estimates” can be replaced with “Pairwise Fst values”). Figure 4 legend … frequencies of what?

**Discussion:**

- Line 277: Distinct dispersal pattern of the populations is not clear how this was obtained
- Line 278: Genetic distance is not estimated in this study and not cited the reference too
- Lines 278-279: The statement needs revision
- Lines 282-283: “…combined with **evidence** of gene flow” that is over statement of the Fst results
- 284-288 & 290-291: needs rephrasing. The references cited have observed high gene flow and low differentiation but not analyzed that it is due to high gene flow and ecological adaptability. Place the reference properly and rephrase the sentence
- Line 297 : seen in “other vectors” but the cited references are on the same species *Ph. argentipes*
- Several paragraphs/sentences are too long,
- Too much explanation of mismatch distribution and BSP. It can be trimmed and made relevant to the observations only
- Statements such as “distinct dispersal patterns”, “substantial gene flow”, and “increased flight capabilities due to wind gusts” should be mentioned appropriately
- Contradictory statements in Lines 282–288 and 315–318

**Conclusion:**

Reword to avoid unverified claims. Instead of “played a pivotal role in propelling leishmaniasis toward epidemic state”, stick to genetic evidences generated in this study.

**Minor Comments and Suggestions:**

Line 46: “Geographic distribution” should be “geographic distance”.

Line 48: Replace “across Sri Lanka” with “among sampled populations”.

Line 49: Clarify “vector-parasite interaction”.

Line 51: “Consistent genetic homogeneity” needs clarification.

Line 80: Replace “marginally explored” with “limited studies exist”; cite existing work.

Line 99–104: Paragraph seems misplaced and overly speculative.

Line 106: “**plausible** scenario”.

Line 107: Rephrase “historical analysis of recent descendants’ molecular data” – e.g., “analysis of extant populations to infer demographic history”.

Line 146: “Linearized Fst”

Line 151: Include software version with the name.

Line 230: “Frequencies of what?” – clarify in Figure 4 legend.

Lines 324–359: Entire paragraph should be shortened and focused on interpretation rather than listing all possible models.
