# Supplementary material for: Demographic history and population structure of Phlebotomus argentipes (Diptera: Psychodidae) complex, the leishmaniasis vector in Sri Lanka
Source: PLoS One. 2025 Dec 2;20(12):e0337428. doi: 10.1371/journal.pone.0337428 (PMC12671780; doi:10.1371/journal.pone.0337428)
Supplement: Attachment — Submitted filename: Response_to_reviewer_comments_auresp_2.docx [file pone.0337428.s007.docx]

**Responses to the editor’ comments**

| Section | Comments | Response |
| --- | --- | --- |
|  | The revised manuscript along with response to reviewers comments was shared to all the reviewers of the original ms. All the reviewers appreciated the efforts made to address many of the previous comments. The ms has improved greatly. However, one of the reviewers has also raised a number of important issues still require further attention. I would request the authors to focus on the comments of Reviewer 3 while revising the ms, particularly focusing on the comments related to Methodology and Discussion. | We sincerely thank the Editor for the valuable feedback and for highlighting the need to further refine the methodology and discussion based on Reviewer 3’s suggestions. We have carefully addressed all the concerns raised by Reviewer 3 with particular attention to the methodological details and interpretation of results in the Discussion section. The manuscript has been thoroughly revised to reflect these improvements. All changes are highlighted in the revised version, and detailed responses to each comment have been provided below. |
|  | Further, I would request the authors to revise the Discussion (i) on the basis of discrepancy in expansion timing between ND4 (10,000-13000 years) and COI (50000 years) and its implications for *P. argentipes expansion, and* (ii) omitting repeat statemen | We appreciate the Editor’s guidance regarding the need to refine the Discussion section. Accordingly, the Discussion has been revised to (i) address the observed discrepancy in population expansion timing inferred from ND4 (10,000–13,000 years) and COI (approximately 50,000 years), including possible biological and evolutionary explanations, and (ii) remove repetitive statements to ensure clarity and conciseness. These revisions have been incorporated in the updated Discussion section (Lines 55-57 in the revised manuscript). |

**Responses to the reviewers’ comments**

**Reviewer #2**

| Section | Comments | Response |
| --- | --- | --- |
|  | The manuscript has improved and is much clearer now. | We sincerely thank the reviewer for their positive feedback and appreciation of the improvements made to the manuscript. |
|  | One element that did come through. Have the authors considered comparing different collection methods? i.e., just cattle baited traps. This may eliminate some differences in species behaviour / sibling species. | We thank the reviewer for this valuable observation. In the present study, four different collection methods cattle-baited traps, manual collections, sticky traps, and light traps were employed across sampling sites to ensure comprehensive representation of *P. argentipes* s.l. populations. The use of multiple standardized trapping methods helped to minimize potential bias arising from differences in species behavior or sibling species composition. As the primary focus of this study was population genetic structure and demographic history, detailed comparisons among trapping methods were beyond its scope. However, we agree that future investigations focusing on trap efficiency and behavioral variation would further enrich the understanding of *P. argentipes* ecology in Sri Lanka. |

**Reviewer #3**

| Section | Comments | Response |
| --- | --- | --- |
|  | The authors have revised the manuscript “Demographic history and population structure of Phlebotomus argentipes (Diptera: Psychodidae) complex, the leishmaniasis vector in Sri Lanka” in response to the earlier review round. I acknowledge the substantial effort made to address the prior comments, and many issues have been improved. However, a number of important points still require further attention before the manuscript can be considered ready for publication. | We sincerely thank the reviewer for recognizing our efforts to improve the manuscript and for the constructive feedback provided throughout the review process. We have carefully re-evaluated all remaining comments and have made further revisions to address each point in detail. The revised manuscript has been strengthened accordingly, particularly in the sections related to methodology, data interpretation, and discussion. |
| Abstract | Line 41: The stated aim should be consistent: “to investigate demographic history and population genetic structure.” | Thank you for your comment. The correction has been made in the revised manuscript, line 41. |
|  | The concatenated dataset is reported in the results but not mentioned in the methods description. Please correct this. | Thank you for your comment. The correction has been made in the revised manuscript, lines 45-47. |
|  | The Mantel test showed only weak, non-significant correlations. Marker-wise reporting adds unnecessary detail. A clearer sentence would be: “Mantel tests showed weak, non-significant correlations between genetic and geographic distance, indicating no evidence of isolation by distance.” | Thank you for your comment. The correction has been made in the revised manuscript, lines 51-53. |
|  | Lines 51–54: The statement is too long and should be simplified for clarity. | Thank you for your comment. The correction has been made in the revised manuscript, lines 57-59. |
|  | Line 54: The focus is only on ND4 results, but a conclusive summary should be drawn from the overall BSP analysis, including both COI and ND4. | Thank you for your comment. The correction has been made in the revised manuscript, lines 55-57. |
|  | Line 55: This sentence appears incomplete and does not fit the context. Please revise. | Thank you for your comment. The revision has been made in the corrected manuscript, lines 57-59. |
| Methods | While the authors now note that COI and ND4 data were reused from a previous study, the Methods section still describes sample collection and DNA extraction in detail, giving the impression that new experimental work was conducted. This is misleading. Please rewrite this section to explicitly state that no new laboratory work was performed, and that the dataset was reused exclusively for additional analyses. | We appreciate the reviewer’s valuable comment. We have now revised the *Materials and Methods* section to clearly state that no new laboratory work was conducted for this study. The correction has been mad in lines 115-128. |
|  | Please remove the STRUCTURE analysis. I regret suggesting its inclusion in the previous round. Upon re-evaluation, STRUCTURE is not appropriate for mitochondrial markers (due to their haploid, maternally inherited, non-recombining nature). Its inclusion is methodologically unsound and should be omitted from the manuscript. | We thank the reviewer for the clarification. We fully agree that STRUCTURE analysis is not appropriate for mitochondrial datasets due to their haploid, maternally inherited, and non-recombining nature. In fact, this analysis was not part of our original submission, as we were aware of these methodological constraints and had initially focused on analyses better suited to mitochondrial data (e.g., haplotype-based and population differentiation indices). The STRUCTURE analysis was incorporated only in response to the reviewer’s earlier suggestion during the first revision. As advised now, we have removed this section and all related figures and references from the manuscript. |
| Results | Lines 197–198: Table 1 does not present average pairwise differences. Please correct the description. | Thank you for your comment. The correction has been made in the revised manuscript, line 180. |
|  | Table 1 legend: Remove the word “separately.” | Thank you for your comment. The correction has been made in the revised manuscript, lines 187. |
|  | Line 200: Add “respectively” at the end of the sentence. | Thank you for your comment. The correction has been made in the revised manuscript, lines 183. |
|  | Line 217: The Mantel test description highlights only ND4, but similar values were obtained for the concatenated dataset. Please be consistent. | We thank the reviewer for pointing this out. Upon rechecking, we realized that the sign of the correlation coefficient for the concatenated dataset was incorrectly reported. The corrected Mantel test results are: COI, r = –0.038 (p = 0.48); ND4, r = 0.040 (p = 0.52); concatenated, r = –0.043 (p = 0.43). None of the correlations are statistically significant, indicating no isolation by distance among populations. The text has been revised accordingly to reflect these corrected values and to consistently indicate that no dataset shows a significant. The correction has been made in the revised manuscript, lines 209 |
|  | Line 270: The Fst values appear to be inappropriately interpreted. Please review carefully. | Thank you for your comment. The correction has been made in the revised manuscript, lines 243. |
|  | Lines 280–284: The description of the mismatch distribution analysis is unclear and should be rewritten for clarity. | Thank you for your comment. The unclear part was rewritten in revised manuscript lines 252-258. |
|  | Lines 285–292: The BSP results should be presented marker by marker (COI and ND4) before providing a combined conclusion. Currently, COI is overlooked. | Thank you for your comment. The correction has been made in the revised manuscript, lines 259-262. |
| Discussion | Paragraph 2: Please expand discussion on the species complex issue and how it may affect the interpretation of results. | Thank you for your comment. The correction has been made in the revised manuscript, lines 295-300. |
|  | Lines 323–325: Fst values are consistently <0.027 across all populations. It is not correct to suggest different dispersal behaviors in certain populations. This statement needs revision. | Thank you for your comment. The correction has been made in the revised manuscript, lines 301-317. |
|  | Lines 323–324: The paragraph contains several general statements, making it confusing. Please rewrite more clearly. | The correction has been done in the revised manuscript. |
|  | Line 332: References appear misplaced; check and correct. | Thank you for your comment. The correction has been made in the revised manuscript, lines 323. |
|  | Line 335: The discussion focuses only on ND4-based expansion timing (~10,000–13,000 years) while COI indicates an older signal (~50,000 years). This discrepancy should be discussed, particularly regarding its implications for P. argentipes expansion. | Thank you for your comment. The correction has been made in the revised manuscript, lines 326- 336. |
|  | Lines 340–342: The sentence is unclear and requires revision. | Thank you for your comment. The correction has been made in the revised manuscript, lines 339-343. |
|  | Lines 343–344: The phrase “possible relation” is vague. Please specify what relationship is being discussed. | Thank you for your comment. The correction has been made in the revised manuscript, lines 342-345. |
| General Remarks | The conclusion that ND4 is the most informative marker should be highlighted and discussed in the main text, not only in passing. | Thank you for your comment. The correction has been made in the revised manuscript. |
|  | Overall, the Discussion is too lengthy in places and includes general or repetitive statements. It should be revised to be more concise, focusing on the observations and their implications. | The correction has been done in the revised manuscript. |
|  | Ensure species names are written consistently throughout the text. | We appreciate the reviewer’s comment and have carefully checked the manuscript to ensure consistency in species nomenclature. The term *Phlebotomus argentipes* s.l. (sensu lato) is used throughout the text to represent the species complex (synonym with *P.argentipes* complex), within which sibling species coexist sympatrically. At its first appearance, the full name *Phlebotomus argentipes* is provided, and the abbreviated form (*P. argentipes* s.l.) is used thereafter in accordance with taxonomic conventions. The same format is consistently applied across text, tables, and figures to maintain clarity and scientific accuracy. |
|  | Expand abbreviations only once, at first mention. | We appreciate the reviewer’s observation. All abbreviations have now been expanded only once at their first mention in the manuscript, following the journal’s style requirement. Previously, abbreviations were defined at the first appearance of each section, However, in accordance with formal writing conventions, full terms were retained at the beginning of paragraphs or sentences to maintain readability and grammatical correctness. |
