# Supplementary material for: Demographic history and population structure of Phlebotomus argentipes (Diptera: Psychodidae) complex, the leishmaniasis vector in Sri Lanka
Source: PLoS One. 2025 Dec 2;20(12):e0337428. doi: 10.1371/journal.pone.0337428 (PMC12671780; doi:10.1371/journal.pone.0337428)
Supplement: Attachment — Submitted filename: Response to reviewer comments.docx [file pone.0337428.s006.docx]

**Responses to the editor’ comments**

| Section | Comments | Response |
| --- | --- | --- |
|  | 1. Please ensure that your manuscript meets PLOS ONE's style requirements, including those for file naming. The PLOS ONE style templates can be found at  <https://journals.plos.org/plosone/s/file?id=wjVg/PLOSOne_formatting_sample_main_body.pdf> and  [https://journals.plos.org/plosone/s/file?id=ba62/PLOSOne_formatting_sample_title_authors_affiliations.pdf](https://track.editorialmanager.com/CL0/https:%2F%2Fjournals.plos.org%2Fplosone%2Fs%2Ffile%3Fid=ba62%2FPLOSOne_formatting_sample_title_authors_affiliations.pdf/1/010f0197fa7f060c-82401d25-4524-43a6-bef0-9b0537dbd73b-000000/Cr91KJ536DfDbBKYJflX9JeAEDjTkosncVYI0_LRVt0=219) | Thank you for your comment. We have carefully revised the manuscript to ensure it adheres to PLOS ONE’s style requirements, including file naming conventions. The manuscript and all supporting files have now been renamed and formatted according to the PLOS ONE style templates. |
|  | 2. We note that Figure 1 in your submission contain [map/satellite] images which may be copyrighted. All PLOS content is published under the Creative Commons Attribution License (CC BY 4.0), which means that the manuscript, images, and Supporting Information files will be freely available online, and any third party is permitted to access, download, copy, distribute, and use these materials in any way, even commercially, with proper attribution. For these reasons, we cannot publish previously copyrighted maps or satellite images created using proprietary data, such as Google software (Google Maps, Street View, and Earth). For more information, see our copyright guidelines: <http://journals.plos.org/plosone/s/licenses-and-copyright>. | Thank you for your comment. The map has been removed from the revised manuscript, and the relevant GPS values are already provided within the current study. As this work is an extension of Wedage et al., 2023, it was not necessary to include the map again. This also resolves the copyright concern raised by the editor. |
|  | 3. If the reviewer comments include a recommendation to cite specific previously published works, please review and evaluate these publications to determine whether they are relevant and should be cited. There is no requirement to cite these works unless the editor has indicated otherwise. | Thank you for the suggestion. We have carefully reviewed the recommended publications. After evaluation, we believe the following references are relevant and have been appropriately cited in the revised manuscript to support and strengthen the context of our findings. |

**Responses to the reviewers’ comments**

**Reviewer #1**

| Section | Comments | Response |
| --- | --- | --- |
|  | Manuscript was well written. Results were interpreted well. They can try with other markers for more variation may be with microsatellites or SSRs. More the number of markers more variance. The above will help have a strong support. | Thank you for your positive feedback and valuable suggestion. We agree that incorporating additional nuclear markers such as microsatellites or SSRs can provide higher resolution and broader genetic variation. However, the current study was focused specifically on mitochondrial DNA regions as part of the originally defined scope and available resources. We acknowledge the value of expanding the marker set and have included this as a recommendation for future studies in the revised version of the manuscript. |

**Reviewer #2**

| Section | Comments | Response |
| --- | --- | --- |
|  | It is good to see papers attempting to tackle the genetics of sand flies as there is a dearth in the data and information currently available. There is still a number of basic gaps in our understanding of sand fly behaviour that needs to also be filled both of which will support the control of leishmaniasis. | Thank you for recognizing the relevance of our work in contributing to the limited genetic data available for sand flies. We fully agree that there are still significant gaps in our understanding of sand fly behaviour, and addressing both genetic and behavioural aspects is essential for developing effective vector control strategies against leishmaniasis. We hope that our findings provide a foundation for future integrated studies in this area. |
|  | While cutaneous leishmaniasis is a major issue in Sri-Lanka, it may be worth mentioning the spread of visceral leishmaniasis as well, which is also caused by L. donovani. Note P. argentipes being the main vector of VL in India, Nepal and Bangladesh, has been controlled with IRS so these countries have reached near elimination of leishmaniasis. Noting Sri-Lanka’s own success with IRS and malaria elimination. | We thank the reviewer for highlighting the importance of visceral leishmaniasis (VL) in neighboring regions. While L. donovani causes both VL and cutaneous leishmaniasis (CL) in Sri Lanka, VL remains extremely rare, with only sporadic cases reported. Our study focuses on CL, the dominant form in the country. |
|  | Ln 99 – it is agreed that that insecticide use can drive resistance in sand flies, however, it is interesting that while kdr is reported as widespread in India sand flies, the level of resistance to pyrethroids was low allowing pyrethroids to be used successfully. Linking this to survival in different habitats is a challenge. There are also some migrant workers in sri-lanka etc, could they be responsible for movement of vector and disease? Sand-flies are also associated with animals that may move larger distance and recent publications show dogs may be a reservoir in India for donovani. | Thank you for your valuable comment. We agree that while insecticide use can drive resistance, it may not fully explain the expansion and survival of *P. argentipes* populations. We have revised the relevant section to more clearly reflect the complexity of resistance patterns, particularly in relation to *kdr* findings from India, and have incorporated a broader perspective that includes potential contributions from human and animal movement. These additions help clarify the multifactorial nature of vector and disease spread in the Sri Lankan context (Line 93-98). |
|  | Line 75 – there is a suggestion of sand flies migrating – as they move short distances, typically 100’s of meters more by hoping etc. This is then picked up in the discussion as how they don’t, but maybe they use the wind. This does not feel well structured or concise. Could this expansion have taken a long time to occur | Thank you for your observation. We acknowledge that the original paragraph lacked clarity in describing the dispersal mechanisms and timescale of sandfly expansion. We have now revised the section to better distinguish between short-range dispersal, gradual population expansion, and potential long-distance dispersal (e.g., wind-assisted or anthropogenic). We also clarified that such expansion likely occurred over an extended period through multiple generations, rather than through rapid long-distance migration. The correction had been made in revised manuscript (Line 98, Line 352-360) |
|  | Methods, a concern here is that the work is completed on P. argentipes s.l. which is a complex of species, that also have potentially different vectorial capacities. Within the complex there may be heterogeneity of gene flow. Is there a reason that species were not identified? This needs to be added in the discussion | We appreciate the reviewer’s important observation regarding the need for species-level resolution within the P. argentipes complex. While mtDNA markers (e.g., COI, ND4) are widely used for population studies, we acknowledge that they may not always differentiate cryptic species with confidence. In Sri Lanka, however, P. argentipes s.l. siblings exhibit overlapping distributions and similar ecological behaviors, reducing the immediate impact of species-level divergence on our broader population genetic conclusions.  Given the challenges in both morphological and molecular differentiation of these cryptic species—as well as the difficulty in obtaining sufficient samples for comprehensive analysis, our study focused on the dominant local population. We have clarified this limitation in the Discussion (Lines 319-327, 344- 350, 417-424) and agree that future work incorporating nuclear markers or high-resolution genomics could provide finer-scale insights. |
|  | This manuscript uses the same samples that were analysed in Ref 16 – also focused on COI and ND4, is the amplified fragments analysed the same ones – this is not clear. What actual lab work was carried out here needs to be clarified. Unfortunately, I am unable to comment on the statistics here. | Thank you for your comment. We confirm that the current study uses the same samples and sequence data (COI and ND4 regions) as presented in our previous publication (Ref [16]), which focused primarily on haplotype diversity and genetic variation across populations of *Phlebotomus argentipes* s.l. in Sri Lanka.  The current manuscript builds upon that foundational work by applying a new set of population genetic analyses, including [e.g., AMOVA, pairwise Fst > neutrality tests, Mantel test, mismatch distribution, and demographic inference], which were not included or discussed in the previous paper. Our aim here is to expand the interpretation of the population structure and demographic patterns using the same sequence data in a broader analytical context.  To avoid confusion, we have now revised the Methodology and Discussion sections to clearly state the relationship between the two studies and to emphasize the novelty of the analytical approach presented in this manuscript. |
|  | Line 288 while the population may show signs of adaptability, which may explain the lack of understanding of the sand fly ecosystem – they are not a mobile species – how do the authors explain this part. | Thank you for the comment. We acknowledge the concern regarding the mobility of *P. argentipes*, which is indeed limited compared to many other insect vectors. The original statement was intended as a general remark about insect adaptability and not specifically about sand flies. To avoid this confusion distinction and added possible explanations relevant to *P. argentipes* (line 319-334). |
|  | 293 is the diverse environmental conditions due to this being a group – would each actual species fit into an environmental condition and with that understanding the competent vectors environment could drive control practices. | Thank you for your thoughtful comment. We agree that understanding species-level ecological adaptation is important when working with cryptic species complexes. However, in the case of *P. argentipes s.l.* in Sri Lanka, all identified sibling species are found to co-exist sympatrically within the same populations, without clear ecological or spatial separation. This overlapping distribution suggests that the observed adaptability to diverse environmental conditions is a shared trait across the sibling species, rather than the result of niche partitioning among them.  While this complicates species-specific vector control, it also highlights the importance of understanding genetic structure and adaptability at the complex level, especially when designing broad-scale intervention strategies. We have clarified this point in the revised Discussion (Line 319-334). |
|  | 305 – this again maybe a species event and it would be good to identify the samples as such. | Thank you for your insightful comment. We did identify the sibling species within the *Phlebotomus argentipes* complex. However, our data show that these cryptic species co-exist sympatrically within the same populations and environmental settings, including at the Hambantota and Medirigiriya sites. Therefore, the observed genetic differentiation between populations is unlikely to be driven by differences in species composition across locations. Instead, it reflects intraspecific population-level variation within the sympatric species complex. We have clarified this point in the Discussion to emphasize the sympatric nature of the sibling species and its implications for interpreting genetic differentiation. |

**Reviewer #3**

| Section | Comments | Response |
| --- | --- | --- |
|  | The manuscript titled "Population expansion pattern of Phlebotomus argentipes (Diptera: Psychodidae) complex, the leishmaniasis vector in Sri Lanka" presents population genetic analyses based on COI and ND4 sequence data generated in their previous study. The data and observations are indeed interesting and have considerable importance for understanding vector dynamics in Sri Lanka. However, in its current form, the manuscript does not effectively convey the true value and significance of the findings. Several major issues need to be addressed to improve the clarity, scientific rigor, and impact of the manuscript.  1. Many sections of the manuscript are unnecessarily lengthy and deviate from the core data and analyses 2. Several statements are exaggerated and should be revised to reflect a more balanced interpretation of the results. 3. The manuscript contains redundant and sometimes contradictory statements that compromise its logical flow and coherence. 4. Some of the cited literature is not directly relevant to the study and should be revised 5. At several places in the manuscript, the flow of ideas is unclear, making it difficult to understand the context or relate certain statements to the preceding content. | We sincerely appreciate the reviewer’s thoughtful and detailed feedback on our manuscript. In response, we have thoroughly revised the manuscript to enhance its clarity, focus, and scientific rigor. Lengthy and tangential sections have been condensed or removed to better emphasize the core data and analyses. We carefully moderated statements that previously appeared exaggerated to provide a more balanced interpretation of our findings. Redundant and contradictory content has been eliminated to improve the logical flow and coherence throughout the manuscript. Additionally, we reviewed the cited literature and updated references to ensure they are directly relevant to the study. Finally, the overall organization and flow of ideas have been refined to improve readability and contextual understanding. We believe these improvements substantially strengthen the manuscript and more effectively convey the significance of our work. |
|  | The manuscript presents potentially valuable findings, but it requires substantial restructuring, elimination of speculative statements, tightening of narrative flow, and a more critical and scientifically sound discussion of the results.  **I have listed here my observations for consideration:**  The title could be revised for clarity and broader relevance.  Suggest using:  “Demographic history of *Phlebotomus argentipes* (Diptera: Psychodidae), the leishmaniasis vector in Sri Lanka” or “**Population dynamics**…” | Thank you for your insightful comment and the manuscript had been revised based on the comments and suggestions. We have changed the title line 1. |
| Abstract | The abstract should clarify that data were reused from a previous study. | Thank you for the comment. We have revised the abstract to clearly state (line 42) that the COI and ND4 sequence data used in this study were reused from our previously published work. |
|  | Statements such as “across Sri Lanka” are misleading given that only five sites were sampled and out of which two showed high Fst values. | Thank you for the helpful comment. We agree with your concern and have revised the sentence to more accurately reflect the meaning (Line 52) |
|  | “**consistent** genetic homogeneity” consistent can be removed | Thank you for the suggestion. We agree that the word “consistent” is unnecessary and have removed it to improve clarity (line 57). |
|  | Lines 43-44: “genetically homogenous population with substantial gene flow across Sri Lanka.” Both homogeneity and gene flow have not been estimated in the study. It is just an indirect observation based on fst values | We agree that direct estimates of gene flow (e.g., migrant tracking) would strengthen our study. However, our inferences are supported by multiple lines of evidence beyond F<sub>ST</sub>:   - PCoA: Minimal clustering among sites (Fig. 4) supports genetic homogeneity. - IBD (Mantel test): No significant isolation-by-distance (Fig. 2), suggesting panmixia. - Bayesian inference- No meaningful population subdivision. - Coalescent analysis (Fig. 7): Estimated historical gene flow   While these methods are indirect, their consistency strongly suggests gene flow and homogeneity. |
|  | Line 46: add linearized before (Fst/1-FSt) | Thank you for your comment. In the revised manuscript (line 49), we have clarified the description as 'genetic and geographic distance' to give a more meaningful idea (Line 49). |
|  | Line 49: “which may reflect vector-parasite interaction” is not clear | Thank you for your comment. In the revised manuscript, this unclear part was paraphrased with disease epidermicity (Line 56). |
|  | Concluding statements need to repharsed and should only be based on the genetic evidence generated from this study | Thank you for your comment. The correction has been made in the revised manuscript, lines 56-59. |
|  | Write clearly a line or two about the analysis carried out (like pairwise fst, Mantle test and mismatch distribution and bayseian skyline plot analysis) and then briefly include the observations from each analyses followed by concluding statement based on the observations. | The correction has been made for the abstract in the revised manuscript, lines 45-47. |
| Introduction | Lines 60–62, 71–72: Multiple citations are used to support claims that are either weakly linked or unverified in those references | Thank you for your comment. The corrections have been made in the revised manuscript (line 66). The cited literature on the taxonomy of P. argentipes has been retained, as it is considered a crucial reference for this study (line 74). |
|  | Some general statements are either not novel or not necessary (Line 83). | The correction has been done in the revised manuscript. |
|  | Phrases such as “historical analysis of recent descendants’ molecular data” are ambiguous and need clearer scientific language. | The correction has been done in the revised manuscript (line 105). |
|  | Line 83: This is general statement and the reference cited has not analyzed this. | Thank you for your comment, and the sentence has been removed from the revised manuscript. |
|  | Structure and flow need significant improvement to clarify rationale and research gap. | The revised manuscript has been improved according to the reviewers’ suggestions. |
| Material and Methods | The data were not generated in this study. The first two paragraphs should be condensed into a brief statement referencing the previous work. | Thank you for your comment. The correction has been done in the revised manuscript (lines 113-114). |
|  | Total number of samples and sequences per population should be explicitly mentioned. | Relevant information had been provided with supplementary materials (S1 table). |
|  | Figure 1 is redundant as already information given in previous study | The correction has been done in the revised manuscript, and the figure has been removed. |
|  | Line 113: “hence” is changing the meaning of the sentence | Thank you for your suggestion, and the correction has been done in the revised manuscript (line 109). |
|  | Suggest performing additional analyses such as STRUCTURE, concatenated gene analysis of COI and ND4, increasing permutations for Fst estimation (Line 147) and the analysis should also be carried out for the pooled sequences from all populations for comparison. | The suggested analysis has been done in the revised manuscript (line 146-155). |
| Results | Initial nucleotide composition (Table 1) and base content discussion seem unnecessary. Suggest replacing this with some relevant information like population-wise sequence numbers, geographic distance between them, diversity indices (citing previous study). | Thank you for the suggestion. The correction has been done in the revised manuscript (Table 1- Line 190). |
|  | Lines 184-185: pairwise differences has not been estimated in this study and please replace "examined localities" with "study populations"). | The correction has been done in the revised manuscript (197-200). |
|  | Check highest Fst values, Table 2 suggests MIR–BAL for ND4, not MED–HAM. | The correction has been done in the revised manuscript (line 200- 201) |
|  | Provide correlation coefficients and p-values with Mantel test (Line 191). | Thank you for your comment. The correction has been done in the revised manuscript (Line 225-228). |
|  | Table 2 and figure 4 legends require better wording (e.g., Table 2 legend: “Genetic differentiation estimates” can be replaced with “Pairwise Fst values”). Figure 4 legend … frequencies of what? | Thank you for your suggestion. The correction has been done in the revised manuscript (line 203). |
| Discussion | Line 277: Distinct dispersal pattern of the populations is not clear how this was obtained | The correction has been done in the revised manuscript (lines 319-334). |
|  | Line 278: Genetic distance is not estimated in this study and not cited the reference too | The correction has been done in the revised manuscript (line 338). |
|  | Lines 278-279: The statement needs revision | The correction has been done in the revised manuscript (line 338). |
|  | Lines 282-283: “…combined with **evidence** of gene flow” that is over statement of the Fst results | The correction has been done in the revised manuscript (line 338). |
|  | 284-288 & 290-291: needs rephrasing. The references cited have observed high gene flow and low differentiation but not analyzed that it is due to high gene flow and ecological adaptability. Place the reference properly and rephrase the sentence | The correction has been done in the revised manuscript (line 319-334). |
|  | Line 297 : seen in “other vectors” but the cited references are on the same species *Ph. argentipes* | The correction has been done in the revised manuscript (line 338-339). |
| **Discussion** | Several paragraphs/sentences are too long, Too much explanation of mismatch distribution and BSP. It can be trimmed and made relevant to the observations only | The correction has been done in the revised manuscript. |
|  | Statements such as “distinct dispersal patterns”, “substantial gene flow”, and “increased flight capabilities due to wind gusts” should be mentioned appropriately | The correction has been done in the revised manuscript. |
|  | Contradictory statements in Lines 282–288 and 315–318 | The correction has been done in the revised manuscript (line 319- 325) |
| **Conclusion** | Reword to avoid unverified claims. Instead of “played a pivotal role in propelling leishmaniasis toward epidemic state”, stick to genetic evidences generated in this study. | The correction has been done in the revised manuscript. |
| **Minor Comments and Suggestions** | Line 46: “Geographic distribution” should be “geographic distance”. | The correction has been done in the revised manuscript (line 49). |
|  | Line 48: Replace “across Sri Lanka” with “among sampled populations”. | The correction has been done in the revised manuscript (line 51). |
|  | Line 49: Clarify “vector-parasite interaction”. | The statement has been removed from the revised manuscript |
|  | Line 51: “Consistent genetic homogeneity” needs clarification. | The correction has been done in the revised manuscript (line 56-58). |
|  | Line 80: Replace “marginally explored” with “limited studies exist”; cite existing work. | The correction has been done in the revised manuscript (line 88). |
|  | Line 99–104: Paragraph seems misplaced and overly speculative. | The correction has been done in the revised manuscript. |
|  | Line 106: “**plausible** scenario”. | The correction has been done in the revised manuscript (line 104-106). |
|  | Line 107: Rephrase “historical analysis of recent descendants’ molecular data” – e.g., “analysis of extant populations to infer demographic history”. | The correction has been done in the revised manuscrip (line 105). |
|  | Line 146: “Linearized Fst” | The correction has been done in the revised manuscrip (line 143). |
|  | Line 151: Include software version with the name. | The correction has been done in the revised manuscript (line 160). |
|  | Line 230: “Frequencies of what?” – clarify in Figure 4 legend. | The correction has been done in the revised manuscript (line 293-294). |
|  | Lines 324–359: Entire paragraph should be shortened and focused on interpretation rather than listing all possible models. | Thank you for your comment. The correction has been done in the revised manuscript. |
